# A case of metachronous cervical and early-stage breast cancer

**DOI:** 10.1007/s12672-025-04042-5

**Published:** 2025-11-29

**Authors:** Iason Psilopatis, Julius Emons, Stefanie Burghaus, Paul Gass, Frederik A. Stuebs, Anja Seibold, Andreas Füller, Matthias W. Beckmann, Patrik Pöschke

**Affiliations:** 1 Department of Gynecology and Obstetrics, Universitätsspital Basel, Basel, Switzerland; 2 Department of Gynecology and Obstetrics, Comprehensive Cancer Center Erlangen-EMN (CCC ER-EMN), Universitätsklinikum Erlangen, Friedrich-Alexander-Universität Erlangen-Nürnberg (FAU), Erlangen, Germany; 3 Department of Gynecology and Obstetrics, Klinikum Chemnitz, Chemnitz, Germany

**Keywords:** Antibody drug conjugate, Breast, Cervical, Cancer, Immunotherapy, Metachronous

## Abstract

**Introduction:**

Cervical and breast cancer are among the most common malignancies in women, typically presenting independently. Their concurrent or metachronous occurrence is rare due to differing etiological factors. This report presents the case of a 53-year-old woman diagnosed with recurrent metastatic cervical cancer who subsequently developed early-stage breast cancer.

**Case presentation:**

A 53-year-old postmenopausal woman with a history of cervical cancer, treated with radical hysterectomy and chemoradiotherapy in 2001, developed a pelvic wall recurrence with distant metastases in 2022. Despite treatment with chemotherapy in combination with checkpoint inhibitor pembrolizumab and subsequent Tisotumab Vedotin, her three-month follow-up in August 2024 revealed a new lesion in the right breast which turned out to be breast cancer. Biopsy confirmed a well-differentiated, hormone receptor-positive, HER2-negative breast cancer with a low proliferation index of 10%.

**Discussion:**

The simultaneous or sequential presentation of cervical and breast cancer is rare, especially under systemic treatment. The patient’s cervical cancer was aggressive, and the subsequent metachronous breast cancer highlights the complex, multifactorial nature of carcinogenesis. The development of a second primary malignancy while a patient is receiving treatment for a metastatic condition is a critical clinical scenario, necessitating individualized treatment strategies, as optimal management remains unclear due to the rarity of such cases.

**Graphical abstract:**

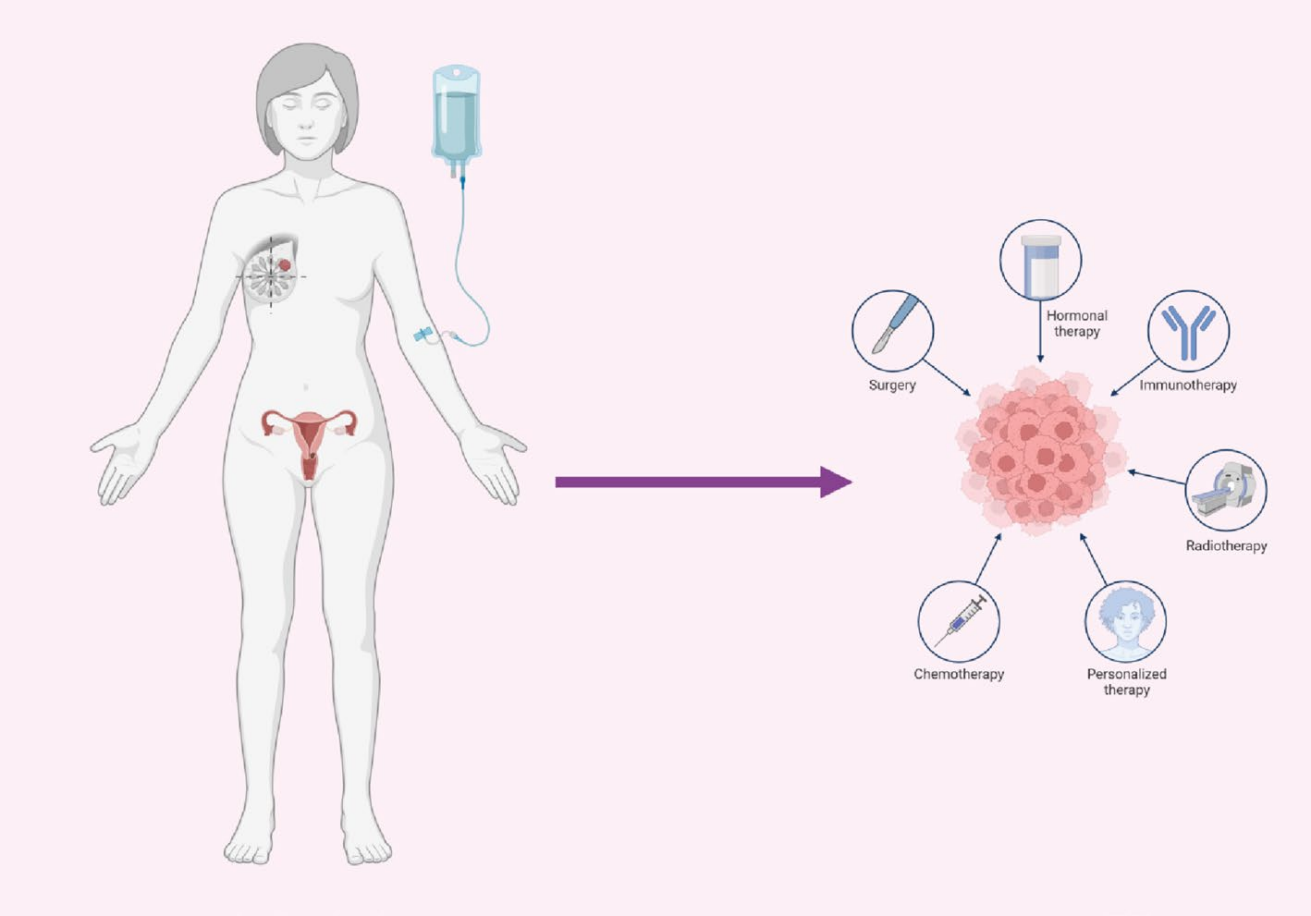

## Research Highlights

- The rare clinical scenario of cervical and breast cancer coexistence.
- The development of breast cancer under Tisotumab Vedotin therapy for cervical cancer.
- The challenges in the management of patients with multiple primary malignancies.

## Introduction

Cervical cancer and breast cancer are two of the most prevalent malignancies affecting women worldwide, each presenting distinct challenges in diagnosis, treatment, and prognosis [1]. While cervical cancer typically arises from the transformation of epithelial cells within the cervix, often related to persistent human papillomavirus (HPV) infection [2], breast cancer develops from the epithelial cells lining the ducts or lobules of the breast [3]. The occurrence of these cancers in isolation is common, but the simultaneous or sequential presentation of both malignancies in a single patient is notably rare and presents unique clinical considerations [4].

In women, breast cancer is often reported alongside ovarian, endometrial, soft tissue, salivary gland, and lung cancer, primarily due to shared etiological factors and genetic syndromes. However, the simultaneous occurrence of breast cancer and cervical cancer is uncommon, likely because the predisposing factors for these two cancers differ significantly [5].

Here, we present the case of a 53-year-old woman who was diagnosed with cervical cancer and subsequently developed metachronous breast cancer.

## Case report

We present the case of a 53-year-old postmenopausal woman (0-Gravida) with a history of metastatic cervical cancer recurrence. She has an Eastern Cooperative Oncology Group (ECOG) performance status of 0 and no additional comorbidities. Family anamnesis was inconspicuous.

The patient’s medical history included a diagnosis of cervical cancer in June 2001, for which she underwent a radical hysterectomy (Wertheim-Meigs procedure). Histology confirmed squamous cell carcinoma of the cervix with bilateral parametrial infiltration, and the tumor was staged as pT3 pN0 (0/26) M0 G4 R0 according to the TNM Classification of Malignant Tumors (TNM) classification system. Adjuvant chemoradiotherapy with six cycles of cisplatin and a total radiation dose of 59.5 Gy was administered between July and August 2001. In February 2022, the patient was diagnosed with a pelvic wall recurrence of cervical cancer, International Federation of Gynecology and Obstetrics (FIGO) stage IVB, with bladder and rectal infiltration, as well as hepatic and pulmonary metastases. A Computer Tomography (CT)-guided biopsy confirmed the recurrence, and Programmed Death-Ligand 1 (PD-L1) testing showed a Tumor Proportion Score of 9%, with < 1% of Immune Cells and a Combined Positivity Score of 9%. From April to September 2022, the patient underwent chemotherapy with carboplatin and paclitaxel in combination with the PD-1 inhibitor pembrolizumab, but treatment was discontinued after eight cycles due to an anaphylactic reaction to carboplatin. An exploratory laparotomy was indicated because of a covered-perforated sigmoid diverticulitis on abdomen CT. Tumor debulking was aborted due to inoperability, and cytology was negative for malignant cells. From November 2022 to February 2023, the patient continued systemic therapy with pembrolizumab monotherapy. In February 2023, imaging showed progressive local recurrence of cervical cancer in the left pelvis, and since March 2023, the patient has been receiving the antibody-drug conjugate Tisotumab Vedotin based on the innovaTV 204 trial (ClinicalTrials.gov identifier NCT03438396).

**Fig. 1:**
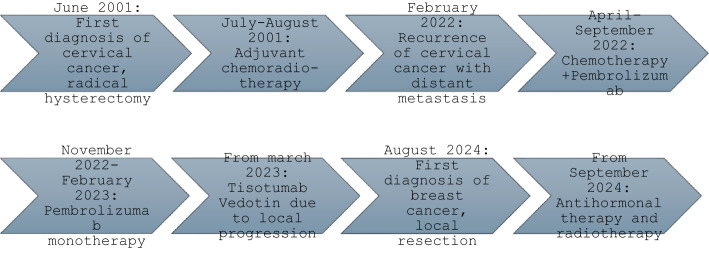
Timeline summarizing the key events

Currently, the patient is stable under treatment with Tisotumab Vedotin, with no evidence of disease progression. The most recent pelvic Magnetic Resonance Imaging (MRI) revealed no signs of new local recurrence after undergoing multimodal therapy for cervical cancer recurrence. However, the recent standard three-month-control CT scan (08/2024) has raised suspicion of a right upper inner quadrant breast carcinoma, staged as cT1 cN0. Apart from this coincidental finding, the CT scan did not show any signs of local recurrence of the metastatic cervical cancer. There was a consistent finding of liver metastasis in segment VIII, and the patient had an unchanged grade IV hydronephrosis on the left side. Breast ultrasound results showed a Breast Imaging-Reporting and Data System (BI-RADS) 5 rating for the right breast, indicating a high suspicion of carcinoma, while the left breast had a BI-RADS 2 rating. Upon right breast biopsy performance for histological examination, the patient was diagnosed with a well differentiated early-stage hormone-receptor-positive, HER2-negative breast cancer and a proliferation index of 10%. As suggested in our interdisciplinary tumor conference, the patient was treated by surgical treatment with breast-conserving segmental resection and axillary sentinel lymphadenectomy. The final postoperative TNM stadium was defined as pT1c pN0 (0/5) L0 Vo Pn0 G2 R0, as such antihormonal therapy and a partial breast radiotherapy were suggested for the adjuvant therapy of the breast cancer. The time interval between the initiation of Tisotumab Vedotin and the discovery of the breast cancer was approximately 17 months (Fig. 1).

## Discussion

The coexistence of cervical and breast cancer in the same patient, particularly when they present as metachronous malignancies, is an exceedingly rare clinical scenario. While breast cancer frequently co-occurs with other malignancies such as ovarian, endometrial, and lung cancers due to shared genetic and etiological factors, the simultaneous or sequential development of cervical and breast cancer is scarcely documented in the literature. This rarity is likely due to the distinct etiological pathways involved in the pathogenesis of these two cancers [4–8].

In this case, the patient’s history of cervical cancer, diagnosed over two decades ago and treated with radical hysterectomy and adjuvant chemoradiotherapy, followed by a recurrence involving the pelvic wall and distant metastases, underscores the aggressive nature of her cervical malignancy. Despite this, the subsequent development of a primary breast cancer highlights the complexity and multifactorial nature of cancer development in this patient.

The literature on synchronous or metachronous occurrences of breast and cervical cancer is sparse, with only a handful of cases reported globally [4, 6–8]. This scarcity of cases makes it challenging to draw definitive conclusions about the optimal management strategies for patients presenting with both malignancies. However, it is crucial to consider the differing biological behaviors, treatment responses, and prognostic factors associated with each cancer type when developing a comprehensive treatment plan [9, 10]. More recently, Kouhen et al. reported on the case of a 66-year-old patient with synchronous cervical and triple-negative breast cancer that received neoadjuvant chemotherapy for breast cancer, followed by breast-conserving surgery and axillary lymph node dissection. Simultaneously, the cervical cancer was addressed with concurrent chemoradiotherapy and brachytherapy, with the patient ultimately showing complete pathological responses in both tumor entities [11]. Similarly, Kumar et al. published the case of a 56-year-old woman with synchronous cervical squamous cell carcinoma and invasive ductal carcinoma of the breast who received chemoradiotherapy, as well as a modified radical mastectomy, resulting in no residual lesion in the breast or axilla, but residual disease in the cervix with need for palliative chemotherapy [12].

In the reported cases, treatment strategies have varied depending on the stage of each malignancy, the patient’s overall health, and the presence of metastases. In our patient’s case, the decision to treat the cervical cancer recurrence with systemic therapy, including the use of Tisotumab Vedotin, followed by the coincidental detection of early-stage breast cancer, required careful consideration of the potential interactions between treatments and the patient’s ability to tolerate multiple lines of therapy. To treat the patient’s breast cancer in curative intention with breast conserving surgery, partial breast radiation and antihormonal therapy with letrozole was chosen because of the good ECOG and most important low interference with the patient’s treatment of her leading condition, the metastatic cervical cancer.

Given the paucity of reported cases, this case contributes valuable insights into the management of patients with concurrent cervical and breast cancer. It underscores the need for heightened vigilance and regular monitoring in patients with a history of one malignancy for the potential development of a second primary cancer. Interestingly enough, patients with primary cervical cancer have been reported to be at risk for developing secondary malignancies including rectal, pancreatic, stomach, cecum, and sigmoid colon cancers [13]. Additionally, it highlights the importance of a multidisciplinary approach to treatment planning, involving oncologists, radiologists, pathologists, and gynecologists, to ensure the best possible outcomes for patients with such complex oncological profiles. In the present case, the patient developed breast cancer under Tisotumab Vedotin therapy for her cervical leading cancer entity. Tisotumab Vedotin represents an antibody-drug conjugate of a monoclonal antibody specific for tissue factor conjugated to monomethyl auristatin E, which targets tissue factor expressing tumors [14]. To date, no reports have been published on secondary carcinomas under Tisotumab Vedotin therapy nor are there any studies investigating the use of Tisotumab Vedotin in breast cancer treatment. As such, the development of breast cancer under Tisotumab Vedotin therapy for cervical cancer cannot be easily pathophysiologically explained (apart from the lifetime risk for breast cancer in women).

In conclusion, the simultaneous occurrence of cervical and breast cancer, as observed in this patient, is a rare phenomenon with few documented cases in the literature. This case not only adds to the limited body of knowledge on this subject but also emphasizes the challenges in managing patients with multiple primary malignancies. Further studies and case reports are needed to better understand the underlying mechanisms, prognostic implications, and optimal treatment strategies for such cases.

## Data Availability

The datasets used and/or analysed during the current study are available from the corresponding author on reasonable request.
