# Correction: A case of metachronous cervical and early-stage breast cancer

**DOI:** 10.1007/s12672-025-04305-1

**Published:** 2025-12-19

**Authors:** Iason Psilopatis, Julius Emons, Stefanie Burghaus, Paul Gass, Frederik A. Stuebs, Anja Seibold, Andreas Füller, Matthias W. Beckmann, Patrik Poeschke

**Affiliations:** 1 Department of Gynecology and Obstetrics, Universitätsspital Basel, Basel, Switzerland; 2 Department of Gynecology and Obstetrics, Comprehensive Cancer Center Erlangen-EMN (CCC ER-EMN), Universitätsklinikum Erlangen, Friedrich-Alexander-Universität Erlangen-Nürnberg (FAU), Erlangen, Germany; 3 Department of Gynecology and Obstetrics, Klinikum Chemnitz, Chemnitz, Germany

**Correction: Discover Oncology (2025) 16:2190** 10.1007/s12672-025-04042-5

In the original publication of this article [1] the author names were transposed. The correct author names are shown in this correction article, the original article has been updated. 
